# Long-term Associations of an Early Corrected Ventricular Septal Defect and Stress Systems of Child and Mother at Primary School Age

**DOI:** 10.3389/fped.2017.00293

**Published:** 2018-01-15

**Authors:** Valeska Stonawski, Laura Vollmer, Nicola Köhler-Jonas, Nicolas Rohleder, Yulia Golub, Ariawan Purbojo, Gunther H. Moll, Hartmut Heinrich, Robert A. Cesnjevar, Oliver Kratz, Anna Eichler

**Affiliations:** ^1^Department of Child and Adolescent Mental Health, University Hospital Erlangen, Friedrich-Alexander University Erlangen-Nürnberg (FAU), Erlangen, Germany; ^2^Department of Psychology, Friedrich-Alexander University Erlangen-Nürnberg (FAU), Erlangen, Germany; ^3^Department of Pediatric Cardiac Surgery, University Hospital Erlangen, Friedrich-Alexander University Erlangen-Nürnberg (FAU), Erlangen, Germany; ^4^kbo-Heckscher-Klinikum, München, Germany

**Keywords:** cardiology, ventricular septal defect, stress, adjustment, cortisol, endocrinology, children, mother

## Abstract

**Introduction:**

Ventricular septal defect (VSD) is the most common congenital heart defect, with larger VSDs typically being corrected with an open-heart surgery during infancy. Long-term consequences of a VSD-corrective surgery on stress systems of child and mother are still unknown. The aim of the present study is to investigate the associations of an early corrected VSD and diurnal cortisol release of child and mother.

**Methods:**

26 children (12 boys) between 6 and 9 years old, who underwent surgery for an isolated VSD within the first 3 years of life, and their mothers participated in the study. Their diurnal cortisol profiles were compared to a sex-, age-, and socioeconomic status-matched healthy control group. Within the VSD group, associations between cortisol and characteristics of surgery and hospitalization were investigated. Child and mother psychopathological symptoms were considered as a possible interfering mechanism of altered cortisol profiles.

**Results:**

Diurnal cortisol profiles of children with an early corrected VSD did not differ from those of controls. However, mothers of affected children exhibited higher cortisol levels in the morning (*p* < 0.001, $\eta_{p}^{2}=0.36$) and a steeper diurnal cortisol slope (*p* = 0.016, $\eta_{p}^{2}=0.17$) than mothers of healthy children.

**Conclusion:**

Results indicate a favorable development of children with an early corrected VSD, in terms of comparable diurnal cortisol profiles with healthy controls, according to a comparable mother-rated psychopathology. Mothers of affected children reveal altered diurnal cortisol levels, without differences in self-rated psychopathology. This divergence should be clarified in future research.

## Introduction

Ventricular septal defect (VSD) is the most common congenital heart defect (CHD), with a worldwide birth prevalence of 2.62 per 1,000 live births (1). Smaller VSDs are able to close spontaneously during the first years of life, whereas larger ones have to be surgically corrected in order to prevent negative health consequences, such as pulmonary hypertension or arrhythmias (2). Most patients with surgically corrected VSD have a comparable life expectancy to the healthy population and are therefore regarded as physically healthy (2). However, despite the good outcome compared to more complex CHDs, VSD diagnosis, surgical correction, and the associated hospitalization may have short- and long-term consequences on the psychological well-being of the child and parents.

Studies from other cohorts that require early-life surgery showed that a pediatric surgery, seen as a life-threatening event in the case of complications and the possibility of permanent impairments, can act as a traumatic event. This may lead to increased symptoms of anxiety and posttraumatic stress disorder in the children and their mothers (3, 4). Children with CHD also showed psychological maladjustment, in terms of more externalizing and especially internalizing symptoms and higher rates of psychiatric disorders (5). However, studies investigating the effects of CHD have mainly combined different defects, with only two studies focusing specifically on VSD. These studies found comparable results for children with an early corrected VSD, with parents reporting more internalizing and externalizing problems in their children than those of healthy controls (6, 7). Regarding parental psychological adjustment, longitudinal studies have shown improvement in mental well-being from the highly stressful preoperative time period until 4 years postoperative (8, 9). However, in the long term, the majority of results indicate a negative impact of CHD on parents in terms of higher stress levels or more symptoms of depression and anxiety than reference groups (10).

Investigating risk factors for maladjustment in children and parents, severity of CHD was mainly related to more negative consequences for psychological adjustment for both the child (5, 11) and its mother (12, 13). Investigating surgery-related factors, associations of child psychological maladjustment with age at surgery (7) and cardiopulmonary bypass duration (6) have been identified. Furthermore, children’s behavioral outcome was reported to be influenced by maternal mental well-being (7, 14), pointing to the importance of maternal health for child outcome.

In general, psychological maladjustment, for example, depressive and anxiety symptoms or high subjective stress levels as multiply described in both children with corrected VSD and their mothers, is associated with dysfunctions of the hypothalamic–pituitary–adrenal (HPA) axis representing the key system of the neuroendocrine stress response (15, 16). An adequate functionality of the HPA axis is essential for an adaptive response to stress, with chronic dysfunctions leading, for example, to an impaired immune system and a higher risk for diseases (17, 18). Thus, examining the HPA axis is important not only for determining current psychological adjustment beyond self-ratings but also for assessing, potentially invisible, long-term risks for later health.

In the context of surgical correction of CHD, only two studies have investigated the short-term effects of a cardiopulmonary bypass surgery on HPA activity in children. They reported a typical stress response to surgery, in terms of increased pre- and perioperative cortisol levels followed by a 48 h postoperative decline (19, 20). Furthermore, associations between cortisol and poorer hemodynamic functions 48 h postoperative were reported, in terms of children with greater atrial filling pressures and a lower cardiac index displaying elevated cortisol levels (20). However, neither long-term consequences nor parental cortisol levels were examined in relation to CHD or more specifically to VSD.

In summary, several studies have investigated the long-term impact of CHD on child and parent psychological adjustment, operationalized by rating scales or clinical interviews (5–10), but none has considered physiological measures of adjustment. Based on the results concerning internalizing and externalizing symptoms and distress in children with CHD and their mothers, accompanying long-term alterations of the HPA axis activity are hypothesized, with a higher severity of CHD corresponding to stronger alterations. Moreover, former studies predominantly combined different defects resulting in a heterogeneous sample group [e.g., Ref. (11, 12, 21, 22)]. In order to better understand the long-term consequences of the surgery of an isolated VSD, the current study extends existing literature about child and parent psychological adjustment to physiological measures. By comparing diurnal cortisol profiles of affected children and their mothers to those of healthy matched controls, the current study aims to investigate long-term associations of early corrected VSD and stress systems of child and mother.

## Materials and Methods

### Study Design and Participants

From 2006 to 2012, 86 children under 3 years of age were operated for a VSD in the Department of Pediatric Cardiac Surgery (University Hospital Erlangen, Germany). In 2015, families were invited for study participation at the Department of Child and Adolescent Mental Health (University Hospital Erlangen, Germany). 25 children had to be excluded from study enrollment due to genetic syndromes (e.g., Down syndrome; *n* = 14), additional congenital malformations (e.g., VACTERL association; *n* = 5), and complex heart defects (e.g., tetralogy of Fallot; *n* = 6). One child died between surgery and start of the study from other than cardiologic reasons, resulting in 60 children fulfilling inclusion criteria. Six families had moved to an unknown address and 15 families were not interested in study participation. Of the 39 participating children (65%), 26 children and their mothers provided valid cortisol samples and were included in the current study. Participating (*n* = 39) and non-participating children (*n* = 21) did not differ in surgery characteristics. Families with valid (*n* = 26) and invalid (*n* = 13) cortisol samples did not differ in sociodemographic, socioeconomic, or surgery characteristics.

In a 2-h session, mothers answered, *inter alia*, standardized questionnaires regarding self and child psychopathology and everyday stress. They were interviewed for socioeconomic data, health, and medication status of herself and her child and were instructed for cortisol sampling at home.

For comparison of diurnal cortisol profiles, a matched case–control study design was used which included in total 52 children and their mothers: 26 healthy controls, who were recruited from the Franconian Cognition and Emotion Studies sample [Erlangen; (23)], were matched for sex, age, and socioeconomic status (SES) to the 26 children (12 boys and 14 girls) who underwent surgery to correct their isolated VSD.

### Measures

#### VSD Surgery Characteristics

Characteristics of the VSD surgery, which dated back 3.8–8.8 years (*M* = 6.2, SD = 1.1) from the current investigation, were routinely recorded in the patient chart of the Department of Pediatric Cardiac Surgery and are presented in Table 1. Surgery characteristics were used as indicators for VSD severity.

**Table 1:** Sociodemographic, health, and surgery characteristics of the sample.

		Total (*N* = 52)	VSD (*n* = 26)	Controls^a^ (*n* = 26)	Ventricular septal defect (VSD) vs. controls		
					*t*/χ^2^	*p*	d/φ
**Sociodemographic characteristics**							

Migration background mother		11 (21.2%)	5 (19.2%)	6 (23.1%)	0.12	0.734	0.05
Migration background father		7 (13.5%)	4 (15.4%)	3 (11.5%)	0.21	0.643	0.07
Education mother (years of education)	<9	0 (0%)	0 (0%)	0 (0%)	3.99	0.136	0.28
	9	14 (26.9%)	10 (38.5%)	4 (15.4%)			
	10	16 (30.8%)	8 (30.8%)	9 (34.6%)			
	13	21 (40.4%)	8 (30.8%)	13 (50.0%)			
Education father (years)	<9	0 (0%)	0 (0%)	0 (0%)	0.95	0.621	0.14
	9	19 (36.5%)	12 (46.2%)	8 (30.8%)			
	10	16 (30.8%)	7 (26.9%)	9 (34.6%)			
	13	16 (30.8%)	7 (26.9%)	9 (34.6%)			
Monthly family income (in €)	<1,000	0 (0%)	0 (0%)	0 (0%)	2.67	0.615	0.23
	1,000–2,000	10 (19.2%)	7 (26.9%)	3 (11.5%)			
	2,000–3,000	20 (38.5%)	8 (30.8%)	12 (46.2%)			
	3,000–4,000	15 (28.8%)	8 (30.8%)	7 (26.9%)			
	4,000–5,000	5 (9.6%)	2 (7.7%)	3 (11.5%)			
	>5,000	2 (3.8%)	1 (3.8%)	1 (3.8%)			
SES		11.13 (2.16)	10.73 (2.32)	11.54 (1.94)	1.36	0.180	0.38
Child sex	Boys	24 (46.2%)	12 (46.2%)	12 (46.2%)	0.00	1.00	0.00
	Girls	28 (53.8%)	14 (53.8%)	14 (53.8%)			
Child age (years)		7.18 (0.77)	7.10 (0.89)	7.27 (0.64)	0.84^b^	0.402	0.23
Mother age (years)		37.47 (5.20)	35.23 (4.83)	39.71 (4.62)	3.41**	0.001	0.95

**Mental health**							

Child psychopathology (SDQ)		9.00 (5.75)	8.73 (5.17)	9.27 (6.38)	0.36	0.739	0.10
Mother psychopathology (BSI)		45.40 (13.88)	44.62 (13.43)	46.19 (14.54)	0.41	0.686	0.11
Mother everyday stress (ESI)		35.13 (9.42)	34.12 (10.46)	36.15 (8.35)	0.78	0.441	0.22
Child medication	Antibiotics	12 (23.1%)	9 (34.6%)	3 (11.5%)	3.90*	0.048	0.27
	Corticosteroids	1 (1.9%)	1 (3.8%)	0 (0%)	1.02	0.313	0.14
	Beta blocker	1 (1.9%)	1 (3.8%)	0 (0%)	1.02	0.313	0.14
Mother medication	Antibiotics	8 (15.4%)	4 (15.4%)	4 (15.4%)	0.00	1.00	0.00
	Corticosteroids	1 (1.9%)	1 (3.8%)	0 (0%)	1.02	0.313	0.14
	Beta blocker	0 (0%)	0 (0%)	0 (0%)	0.00	1.00	0.00

**Surgery characteristics (only VSD)**		***M* (SD)**	**Range**				

Child age at surgery (months)		11.38 (9.67)	2–32				
Duration of surgery (min)		235.9 (45.9)	155–355				
Cardiopulmonary bypass duration (min)		127.5 (37.0)	56–218				
Duration of cardiac arrest (min)		73.42 (30.67)	15–148				
Length of stay in the intensive care unit (days)		4.35 (4.29)	1–16				
Length of hospitalization (days)		9.38 (4.91)	5–24				
Length of surgical scar (cm)		9.63 (1.73)	6.5–15.0				

*Continuous variables are expressed as mean (SD) and tested with independent *t*-tests (df = 50) with Cohen’s *d* indicating effect size. *T*-scores are displayed as absolute values. Categorical variables are expressed as *n* (%) and tested with chi-squared test (χ^2^ varies according to the number of categories of the tested variable between df = 1 and df = 4) with φ-coefficient used as a measure of effect size. SES, socioeconomic family status, additive combination of parental migration background, education level, and the family income, theoretical range 3–16. SDQ, Strength and Difficulties Questionnaire (24), total difficulties score is indicated (theoretical range: 0–40). BSI, Brief Symptom Inventory (25), global severity index is indicated (*T*-score). ESI, Everyday Stressors Index (26), sum-score is indicated (theoretical range: 0–60). Medication, medication intake within the last half year*.

*^a^Controls were matched for child age, sex, and SES*.

*^b^df adjusted for unequal variances based on Levene*.

*p < 0.05.

**p < 0.01.

#### Diurnal Cortisol Profiles

Parents were instructed to collect five saliva samples at home using Salivette sampling devices (Sarstedt, Nümbrecht, Germany). Out of five salivary samples on a single day (T1: at awakening, T2: 30 min after awakening, T3: at 12:00 p.m., T4: at 5:00 p.m., and T5: at bedtime), a diurnal cortisol profile was compiled for both child and mother. In a daily log, mothers documented the awakening and sampling times as well as other variables regarding the day of sampling, such as school day (yes/no), medication intake and diseases, for child and mother, respectively. Mothers were instructed to postpone saliva sampling in acute illness. Child and mother should not have consumed anything by mouth apart from water and should not brush the teeth before the first two samples or directly before the last sample. They were informed not to take food or drink other than water 30 min before each following sample (27). Saliva samples were stored at −20°C after return to the laboratory. Cortisol levels were analyzed with a luminescence immunoassay (ELISA; IBL International, RE56211, Hamburg, Germany). Photometric measurements were conducted with a Multiskan^TM^ GO microplate spectrophotometer (Thermo Fisher Scientific, Vantaa, Finland).

Five parameters were calculated out of cortisol levels of the five samples. The first sample was used as waking cortisol, the last as bedtime cortisol. The cortisol awakening response (CAR) was computed as area under the curve with respect to increase (AUC_I_) from first to second sample (28). Total cortisol release throughout the day was calculated as area under the curve with respect to ground (AUC_G_) including all samples (28). Cortisol decline from awakening to bedtime was represented by the diurnal cortisol slope that was assessed as slope over all samples except the second one.

**Figure 1:**
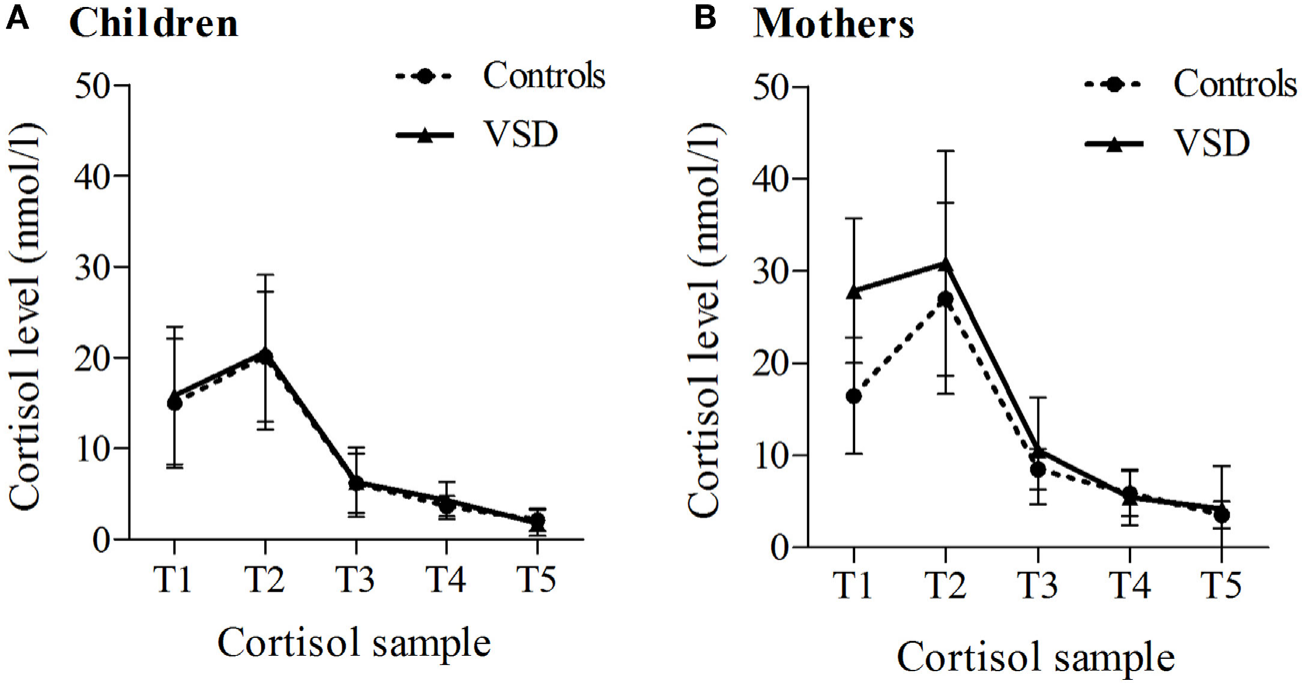
Diurnal cortisol profiles separately for ventricular septal defect (VSD) vs. control group, for children **(A)** and their mothers **(B)**. Mean values and 95% confidence intervals are displayed. Default sampling times: T1 = at awakening, T2 = 30 min after awakening, T3 = 12:00 p.m., T4 = 5:00 p.m., and T5 at bedtime (*n* = 31–49).

Because of medication intake (corticosteroids and beta-blocker), two VSD children and one VSD mother were excluded with their corresponding control partner, resulting in *n* = 48 children and *n* = 50 mothers entered in cortisol analyses. Regarding the sensitive cortisol reaction in the morning (27), single samples that were collected more than 15 min after awakening (children: *n* = 11, mothers: *n* = 15) were interpreted as invalid first samples and excluded from waking, CAR, and diurnal slope analyses. For CAR calculation, second samples that were collected less than 15 min or more than 45 min after awakening were additionally excluded (children: *n* = 15, mothers: *n* = 19). Outliers in cortisol samples, defined as raw values more than three SDs from group mean, were removed (children: *n* = 5, mothers: *n* = 5). In order to improve normal distribution, cortisol raw values were ln-transformed before calculation of diurnal cortisol parameters. Depending on the parameter of interest, a minimum of *n* = 30 and a maximum of *n* = 47 subjects were included in analyses, as indicated in Table 2. Table S1 in Supplementary Material represents the children’s sample size as well as descriptive statistics of raw cortisol values and sampling times; Table S2 in Supplementary Material contains the same information for mothers. Diurnal cortisol profiles, separated into VSD vs. control group and children vs. mothers, are illustrated in Figure 1.

**Table 2:** Effects of an early ventricular septal defect (VSD) surgery on child and mother diurnal cortisol parameters: results of one-way analyses of covariance.

Children	Total sample		VSD		Controls		VSD vs. controls			
	*N*	Mean (SD)	*N*	Mean (SD)	*N*	Mean (SD)	*F*	df	*p*	$\eta_{p}^{2}$
Waking cortisol^a^^,^^b^^,^^c^	35	2.72 (0.41)	17	2.74 (0.41)	18	2.69 (0.41)	0.06	1,30	0.812	0.00
Bedtime cortisol^d^	45	1.02 (0.40)	21	0.95 (0.41)	24	1.07 (0.39)	1.47	1,42	0.232	0.03
CAR^a^^,^^b^	30	0.07 (0.14)	12	0.06 (0.11)	18	0.08 (0.16)	0.03	1,26	0.876	0.00
Total release^c^^,^^d^^,^^e^	45	23.11 (3.44)	21	23.05 (3.68)	24	23.16 (3.30)	0.10	1,40	0.752	0.00
Diurnal slope^d^	35	−0.12 (0.05)	17	−0.12 (0.05)	18	−0.13 (0.05)	0.12	1,32	0.730	0.00

**Mothers**	**Total**		**VSD**		**Controls**		**VSD vs. controls**			
	***N***	**Mean (SD)**	***N***	**Mean (SD)**	***N***	**Mean (SD)**	***F***	**df**	***p***	$\eta_{p}^{2}$

Waking cortisol^a^^,^^b^	35	3.02 (0.42)	15	3.32 (0.31)	20	2.80 (0.35)	16.54**	1,31	<0.001	0.35
Bedtime cortisol^c^^,^^d^^,^^f^	47	1.41 (0.57)	23	1.36 (0.74)	24	1.45 (0.33)	0.60	1,42	0.443	0.01
CAR^a^^,^^b^	30	0.07 (0.12)	12	0.02 (0.06)	18	0.12 (0.14)	1.18	1,26	0.287	0.04
Total release^c^^,^^d^^,^^e^	47	32.14 (6.01)	23	31.45 (5.80)	24	32.79 (6.26)	0.30	1,42	0.586	0.01
Diurnal slope^d^^,^^f^	35	−0.11 (0.05)	15	−0.14 (0.06)	20	−0.09 (0.04)	6.44*	1,31	0.016	0.17

*CAR, cortisol awakening response*.

*Total release = total cortisol release throughout day. Exclusion of participants due to medication intake. Exclusion of T1 samples with >15 min since awakening from analyses of waking cortisol, CAR, and diurnal slope analyses. Exclusion of T2 samples with <15 min or >45 min since awakening from CAR analyses. Models were adjusted for specific covariates for the child and mother cortisol parameters, respectively*.

*^a^Awakening time*.

*^b^Time between awakening and first sample*.

*^c^Mother everyday stress: sum score of the Everyday Stressors Index [ESI; (26)]*.

*^d^Time between first and last sample*.

*^e^School day: yes/no*.

*^f^Child psychopathology: total difficulties score of the Strength and Difficulties Questionnaire (24)*.

*p < 0.05.

**p < 0.01.

#### Potential Covariates

Socioeconomic status, psychopathology of child and mother, and everyday stress were examined as potential covariates. Family SES was calculated based on maternal and paternal migration background [two-staged: migration background yes (0) vs. no (1)], education level [four-staged (1–4): <9, 9, 10, or 13 years of education] and family income per month [six-staged (1–6): <1,000, 1,000–2,000, 2,000–3,000, 3,000–4,000, 4,000–5,000, and >5,000 Euro per month]. SES ranged between 3 and 16, with higher values indicating a higher family status. For assessing child psychopathology, mothers rated the 25-item Strength and Difficulties Questionnaire [SDQ; (24)] on a three-point Likert scale. The SDQ includes four scales regarding mental health problems (Emotional problems, Conduct problems, Hyperactivity/Inattention, and Peer problems), which can be summed to a Total Difficulties Score. For analyses, the Total Difficulties Score was used, values ranging between 0 and 40, with higher scores indicating more problems. Mothers’ psychopathology was measured with the self-rating Brief Symptom Inventory [BSI; (25)]. Patient’s symptoms and their intensity are assessed by 53 items on a five-point Likert scale, which cover nine symptom dimensions (Somatization, Obsessive-Compulsiveness, Interpersonal Sensitivity, Depression, Anxiety, Hostility, Phobic Anxiety, Paranoid Ideation, and Psychoticism). As a measure for global maternal distress, the Global Severity Index (standardized *T*-scores, *M* = 50, SD = 10) was used in analyses, with higher values indicating more psychological distress. Mothers’ level of everyday stress was assessed using the self-rating Everyday Stressors Index [ESI; (26)] consisting of 20 items on a three-point Likert scale. The ESI including five scales (Financial Concerns, Role overload, Employment problems, Parenting worries, and Interpersonal Conflict) assesses common problems faced on a daily basis. For analyses, the sum score was used, ranging between 0 and 60, with higher values representing higher level of everyday stress.

### Statistical Analysis

All analyses were performed with SPSS (version 21, SPSS, Chicago, IL, USA). Group differences between VSD and control group in sociodemographic, health, and surgery characteristics were tested with *t*- and chi-squared tests. Hypotheses were tested for children and mothers separately. For examining group differences in cortisol profiles between the VSD and control group, univariate analyses of covariance (ANCOVAs) were carried out for each of the five cortisol parameters. In order to identify risk factors for cortisol variability within the VSD group, multiple regression models were applied. Surgery characteristics that were significantly associated with a single cortisol parameter tested by Pearson correlation (*r*) were used as predictors; cortisol parameters were used as outcomes in independent models. Covariates were included if they were significantly associated with a cortisol parameter, tested using Pearson correlation (*r*), and then entered in a first step to the model. Effect sizes for ANCOVA results were indicated by partial η^2^ $\left(\eta_{p}^{2}\right)$, with $\eta_{p}^{2}<0.06$ interpreted as small, $0.06\leq \eta_{p}^{2}<0.14$ as medium, and $\eta_{p}^{2}\geq 0.14$ as large effects (29). For all analyses, significance level was set to *p* < 0.05. Adjustment for multiple testing was not applied because of the exploratory nature of the study.

## Results

### Demographic Data

Children were investigated at the age of 6.0–8.8 years (VSD: *M* = 7.1, SD = 0.9; controls: *M* = 7.3, SD = 0.6), their mothers were between 24 and 49 years old (VSD: *M* = 35.2, SD = 4.8; controls: *M* = 39.7, SD = 4.6). In total, 21.2% of the children’s mothers (VSD: 19.2%, controls: 23.1%) and 13.5% of the children’s fathers (VSD: 15.4%, controls: 11.5%) had a migration background. As shown in Table 1, the groups differed in mother age, with VSD mothers being younger than mothers of the control group (*p* = 0.001). There were no differences in child [SDQ (total difficulties score): VSD: *M* = 8.7, SD = 5.2; controls: *M* = 9.3, SD = 6.4] or mother psychopathology [BSI (global severity index): VSD: *M* = 44.6, SD = 13.4; controls: *M* = 46.2, SD = 14.5] or mother-rated everyday stress [ESI (sum score): VSD: *M* = 34.2, SD = 10.5; controls: *M* = 36.2, SD = 8.4] between the groups. Children and mothers of the VSD vs. control group did not differ in corticosteroid and beta blocker medication intake. Children with an operated VSD got more antibiotics during the last 6 months than controls (*p* = 0.048). No mother reported severe somatic diseases for her child or herself. Table 1 summarizes sociodemographic, health, and surgery characteristics of the sample, separated for the VSD and control group.

### Covariates

In order to control for time effects, parameter-relevant time frames were added as covariates to the analyses: awakening time and time between awakening and T1 for waking cortisol and CAR; time between T1 and T5 for bedtime cortisol, diurnal slope, and total release. Further covariates were specifically controlled for, as significant associations differed between cortisol parameters and between children and mothers as presented in Table S3 in Supplementary Material. For both children and mothers, type of day was significantly associated with total cortisol release (children: *p* = 0.002; mothers: *p* = 0.001). On a school day, both exhibited more cortisol throughout the day than on holiday or weekend. Mothers who rated the child psychopathology to be higher had elevated bedtime cortisol levels (*p* = 0.008) and flatter diurnal slopes (*p* = 0.046). Mothers with enhanced self-rated psychopathology and elevated everyday stress had higher bedtime values (*p* = 0.045; *p* = 0.047) and more cortisol release throughout the day (*p* = 0.010; *p* = 0.001). Furthermore, mother-rated everyday stress was associated with higher waking cortisol levels (*p* = 0.042) and more total release (*p* = 0.011) in children. Maternal psychopathology and everyday stress were highly correlated (*r* = 0.719, *p* < 0.001); however, only everyday stress was included as covariate since it was found to explain more of the cortisol variances than maternal psychopathology.

### Child Cortisol

Analyses of covariance revealed no differences between VSD children and healthy controls in either cortisol parameter, as shown in Table 2. Regarding only the VSD children, surgery characteristics that were significantly associated with child cortisol—as presented in Table S4 in Supplementary Material—were tested as predictors of cortisol parameters. Because of large correlations of the duration of surgery with the cardiopulmonary bypass duration (*r* = 0.95, *p* < 0.001) and the duration of cardiac arrest (*r* = 0.89, *p* < 0.001), only duration of surgery was included as a predictor in the regression model. No surgery characteristic predicted cortisol parameters significantly or explained significantly more variance than covariates, as displayed in Table S5 in Supplementary Material.

### Mother Cortisol

As presented in Table 2, mothers from children with a corrected VSD showed significant differences in their diurnal cortisol profiles compared to controls, with large effect sizes $\left(\eta_{p}^{2}=0.17-0.36\right)$. VSD mothers had higher waking cortisol levels (*p* < 0.001) and a steeper diurnal slope (*p* = 0.016). Within the VSD mothers, no single surgery characteristic was associated with any cortisol parameters, as shown in Table S4 in Supplementary Material.

## Discussion

The long-term effects of surgical VSD correction on the diurnal cortisol release of affected children at primary school age and their mothers were investigated here for the first time. No differences in diurnal cortisol profiles were found between children affected by a VSD and healthy controls, representing a favorable result for child development after VSD surgery. Mothers of affected children showed altered parameters, in particular elevated cortisol levels at awakening resulting in a steeper diurnal slope. Diurnal profiles were not influenced by variables related to the surgery or hospitalization.

### Children

Children affected by an isolated VSD, which was corrected within the first 3 years of life, showed comparable diurnal cortisol profiles to healthy controls. The heart defect and its surgical repair were not associated with impaired child stress activity 3–8 years after surgery. Normal HPA axis activity is an important correlate of a positive psychological adjustment and corresponds to the comparable level of mother-reported psychopathology between children with VSD and healthy controls found in the current study. However, in former studies, parents reported more behavior problems in children with various CHDs [e.g., Ref. (11, 30, 31)] and specifically, as investigated in the current study, in VSD (6, 7), due to which a correspondingly dysregulated HPA axis function was originally expected.

The current study included only children without genetic syndromes or additional congenital malformations, resulting in a quite homogenous sample group that was rated as physically healthy by attending cardiologists after surgery. Therefore, the current sample might differ from other VSD cohorts in terms of a higher health status and less chronic impairments resulting in a better psychological and associated physiological adjustment than described for children in former studies (6, 7). A systematic participation bias, in the sense that more families with healthy children chose to take part in the current study, could be a further reason for the diverging results regarding psychopathology. Describing the physical and psychological status of children in more detail is recommended for future studies in order to improve comparability of investigated cohorts and clarify diverging results.

### Mothers

Mothers of children with an early corrected VSD displayed a distinct cortisol profile from control mothers with higher cortisol levels in the morning. No differences were apparent for bedtime cortisol, so consequently a steeper diurnal slope across the day was observed in mothers of affected children. A similar profile with higher morning cortisol levels without differences in evening cortisol was described in patients with anxiety disorders and comorbid depressive disorder (6, 32). Higher cortisol levels have been found in depressed individuals (33, 34). Even though a steeper diurnal cortisol slope was associated with numerous other favorable mental and physical health outcomes and is therefore typically interpreted as salubrious profile, a recent meta-analysis reported a trend toward a steeper diurnal slope, driven by elevated morning cortisol, in patients with anxiety disorders (35). Against this background, cortisol results might be interpreted as the physiological counterpart to the reports of heightened distress and affective problems by the mothers of children with various CHDs found elsewhere (13, 22, 36, 37).

In the current study, however, no differences were observed regarding psychopathology or everyday stress. This is in accordance with a few studies that did not find any differences in self-reported mental well-being (9, 38) or even better psychological adjustment in mothers of children affected by various CHDs (21, 39) and therefore may be interpreted as good adjustment after stressful experiences with the cardiac procedure of their children. Alternatively, altered diurnal cortisol profiles without differences in self-ratings also may indicate adjusted reference norms for perceiving stress and problems in parents. After the stressful event of the VSD diagnosis and surgical correction in the first years of their children’s life, mothers might have adjusted their internal representation of stress and problems, resulting in reports of low distress and a comparable mental well-being to mothers of healthy children. Since no other study has investigated the physiological adjustment of the HPA axis in mothers of children with VSD—or more general CHD—in comparison to the psychological distress, more studies are needed to clarify these hypotheses. It is speculated that, despite comparable psychopathology and stress in self-ratings, altered HPA axis activity found in the current study might indicate an existing, but invisible long-term risk for later mothers’ health. Regarding the importance of maternal mental well-being for psychological adjustment and behavior of children with various CHDs (7, 14, 40), long-term studies are needed in order to get information about developmental effects and the potential impact of altered maternal HPA axis function for later mental and somatic health of mothers and their children.

Interestingly, no surgical characteristic was associated with HPA axis activity within children affected by VSD or their mothers. This might imply that alterations in diurnal cortisol found in mothers correspond to the diagnosis and correction of the VSD as a critical event rather than severity of the heart defect or surgery and hospitalization. This is in accordance to other studies reporting no differences in parenting stress between parents of children with more simple vs. more complex CHD (41, 42), suggesting no significant impact of the severity of CHD. First of all, even mothers of children affected by a VSD, which is frequently rated as mild or moderate form of CHD, showed altered physiological profiles in the current study. Second, no surgery characteristic explained variance in mother cortisol, indicating that the experience of the diagnosis and surgery *per se* is associated with HPA axis alterations.

### Limitations

The current study included a small sample of 26 affected children and their mothers compared to a matched control group. Sample size had to be further reduced because of non-compliance for default sampling time in several cases, especially for the first two samples, resulting in a loss of 11–19 samples for single parameter analyses. Thus, especially analyses regarding surgery characteristics as predictors for cortisol variance have to be regarded as exploratory ones. Additionally, sex effects were not investigated in the current study, due to small sample size. Results have to be replicated in a larger cohort, taking account of possible differences between boys and girls.

Cortisol samples were only collected on a single day, limiting the reliability of cortisol measurements. Cortisol levels might potentially be more influenced by day-specific state than trait factors. Several measurements on multiple days would allow averaging and thereby validation of the diurnal profile and are recommended for future studies. Nevertheless, associations, for example, between mother cortisol release and everyday stress (*r* = 0.47, *R*^2^ = 0.22) indicate that the current results can be interpreted, at least as preliminary findings. Furthermore, samples were collected only once per participant, not allowing analyzing developmental trajectories or causal relationships of the diurnal cortisol alterations. Additionally, it cannot be ruled out that differences in mother cortisol already existed before the VSD diagnosis and surgery; prospective studies are needed to assess this point.

In order to get more specific information about the effects of both VSD and its correcting surgery, it would be interesting to compare affected children and their parents with vs. without surgical repair. Furthermore, by adding a comparison group of children with another CHD, specificity of found alterations to VSD could be clarified.

## Conclusion

The current study describes a favorable, unimpaired adjustment of children with an early corrected isolated VSD in terms of HPA activity compared to healthy controls. Cortisol results are supported by comparable levels of child psychopathology in mother-ratings. However, maternal HPA axis activity was considerably altered which might represent a potential risk factor for later mental and somatic health. Mothers of VSD-affected children showed higher morning cortisol levels and a steeper diurnal slope compared to mothers of healthy controls, although differences were not seen in maternal self-ratings. This discrepancy should be further investigated. Future studies regarding the HPA axis activity are needed in order to validate and explain the underlying mechanisms of the current physiological results and link them to the numerous findings from self-rating assessments. Understanding the psychological and physiological impact of an early VSD-corrective surgery on both child and parents in the long term is important for an adequate support for these families.

## Ethics Statement

The study was approved by the local ethics committee of the Medical Faculty of the University of Erlangen-Nürnberg and conducted in accordance with the Declaration of Helsinki. Written informed consent of the mothers and assent of the children were obtained.

## Author Contributions

VS, LV, NR, YG, and AE analyzed the data and/or interpreted the results. AP, GM, HH, RC, OK, and AE initiated and designed the study. AP and RC were the pediatric consultants. VS, NK-J, and AE supervised the data acquisition. VS and LV drafted the initial manuscript. All the authors reviewed the manuscript and approved the final manuscript.

## Conflict of Interest Statement

The authors declare that the research was conducted in the absence of any commercial or financial relationships that could be construed as a potential conflict of interest.

## Abbreviations

CAR, cortisol awakening response; CHD, congenital heart defect; HPA axis, hypothalamic–pituitary–adrenal axis; SES, socioeconomic status; VSD, ventricular septal defect.
