# Supplementary material for: Long-term Associations of an Early Corrected Ventricular Septal Defect and Stress Systems of Child and Mother at Primary School Age
Source: Front Pediatr. 2018 Jan 15;5:293. doi: 10.3389/fped.2017.00293 (PMC5775274; doi:10.3389/fped.2017.00293)
Supplement: Supplementary file 1 [file table_1.PDF]

Table S1. ***Child diurnal cortisol measures: descriptive statistics of cortisol levels and sampling times of valid samples***

	Total sample		VSD		Controls[a]		VSD vs. controls		
	N	Mean (*SD*)	*n*	Mean (*SD*)	*n*	Mean (*SD*)	*t*(30-45)	*p*	*d*
Cortisol levels (nmol/l)									
T1	35	15.40 (7.25)	17	15.84 (7.58)	18	14.99 (7.13)	0.34	.734	0.12
T2	32	20.34 (7.64)	14	20.62 (8.48)	18	20.12 (7.17)	0.18	.858	0.06
T3	47	6.26 (3.50)	23	6.33 (3.80)	24	6.19 (3.26)	0.14	.889	0.04
T4	47	3.97 (1.66)	23	4.29 (2.07)	24	3.67 (1.10)	1.27[b]	.212	0.37
T5	45	2.00 (1.33)	21	1.83 (1.44)	24	2.15 (1.25)	0.78	.438	0.23
Sampling time information									
Awakening time	47	7:45 (1:01)	23	7:51 (1:00)	24	7:40 (1:02)	0.59[b]	.557	0.17
Time T1	35	7:56 (1:07)	17	8:10 (1:02)	18	7:44 (1:11)	1.14	.264	0.39
Time T2	32	8:22 (1:06)	14	8:31 (1:01)	18	8:15 (1:11)	0.67	.510	0.24
Time T3	47	12:25 (0:46)	23	12:28 (0:51)	24	12:22 (0:42)	0.43	.673	0.13
Time T4	47	17:21 (0:39)	23	17:27 (0:51)	24	17:15 (0:23)	1.01	.320	0.30
Time T5	45	20:21 (0:42)	21	20:28 (0:46)	24	20:14 (0:37)	1.06	.297	0.32
Awakening – T1 (min)	35	5.91 (5.41)	17	7.18 (5.50)	18	4.72 (5.20)	1.36	.148	0.46
T1 – T5 (hours)	47	12.33 (1.15)	23	12.28 (0.99)	24	12.38 (1.31)	0.30	.765	0.09

Note: Default sampling times: T1 = at awakening, T2 = 30 minutes after awakening, T3 = 12 p.m., T4 = 5 p.m., T5 at bedtime. Exclusion of participants due to medication intake. Exclusion of T1 samples with >15 minutes since awakening. Exclusion of T2 samples with <15 minutes or >45 minutes since awakening. *t*-statistics and *p*-values refer to the independent t-test, with *t*-scores displayed as absolute values. Cohen's *d* indicate effect size: *d* = 0.2-0.5 small effect, *d* = 0.5-0.8 medium effect, *d* > 0.8 large effect (1). [a]Controls were matched for child age, sex and SES. [b]*df* adjusted for unequal variances based on Levene. $^{+}p < .10$, $^{*}p < .05$. $^{**}p < .01$.

References

1. Cohen J. Statistical power analysis for the behavioral sciences. Hillsdale, NJ: Erlbaum; 1988.
