# Supplementary material for: Long-term Associations of an Early Corrected Ventricular Septal Defect and Stress Systems of Child and Mother at Primary School Age
Source: Front Pediatr. 2018 Jan 15;5:293. doi: 10.3389/fped.2017.00293 (PMC5775274; doi:10.3389/fped.2017.00293)
Supplement: Supplementary file 2 [file table_2.PDF]

Table S2. ***Mother diurnal cortisol measures: descriptive statistics of cortisol levels and sampling times of valid samples***

	Total sample		VSD		Controls[a]		VSD vs. controls		
	N	Mean (*SD*)	*n*	Mean (*SD*)	*n*	Mean (*SD*)	*t*(29-48)	*p*	*d*
Cortisol levels (nmol/l)									
T1	35	21.36 (8.97)	15	27.87 (7.86)	20	16.47 (6.31)	4.76**	<.001	1.63
T2	31	28.50 (11.07)	12	30.84 (12.16)	19	27.02 (10.39)	0.93	.358	0.34
T3	49	9.49 (4.41)	24	10.51 (5.78)	25	8.52 (2.20)	1.58[b]	.124	0.45
T4	47	5.69 (2.71)	22	5.47 (3.03)	25	5.89 (2.45)	0.53	.602	0.16
T5	47	3.87 (3.42)	23	4.20 (4.69)	24	3.55 (1.45)	0.64[b]	.531	0.19
Sampling time information									
Awakening time	50	7:12 (1:14)	25	7:22 (1:18)	25	7:02 (1:09)	0.93	.355	0.26
Time T1	35	7:34 (1:12)	15	8:09 (0:49)	20	7:07 (1:17)	2.70*	.011	0.92
Time T2	31	8:07 (1:15)	12	8:47 (0:50)	19	7:43 (1:18)	2.49*	.019	0.92
Time T3	49	12:33 (0:54)	24	12:35 (0:58)	25	12:31 (0:51)	0.22	.830	0.06
Time T4	47	17:25 (0:52)	22	17:27 (0:54)	25	17:25 (0:51)	0.13	.898	0.04
Time T5	47	22:05 (1:14)	23	21:51 (1:14)	24	22:19 (1:13)	1.33	.189	0.39
Awakening – T1 (min)	35	6.23 (5.35)	15	8.00 (5.79)	20	4.90 (4.71)	1.75[+]	.090	0.60
T1 – T5 (hours)	47	14.49 (1.79)	23	14.06 (1.27)	24	14.89 (2.10)	2.29*	.027	0.67

Note: Default sampling times: T1 = at awakening, T2 = 30 minutes after awakening, T3 = 12 p.m., T4 = 5 p.m., T5 at bedtime. Exclusion of participants due to medication intake. Exclusion of T1 samples with >15 minutes since awakening. Exclusion of T2 samples with <15 minutes or >45 minutes since awakening. *t*-statistics and *p*-values refer to the independent t-test, with *t*-scores displayed as absolute values. Cohen’s *d* indicate effect size: *d* = 0.2-0.5 small effect, *d* = 0.5-0.8 medium effect, *d* > 0.8 large effect (1). [a]Controls were matched for child age, sex and SES. [b]*df* adjusted for unequal variances based on Levene. [+]$p < .10$, *$p < .05$. **$p < .01$.

References

1. Cohen J. Statistical power analysis for the behavioral sciences. Hillsdale, NJ: Erlbaum; 1988.
