# Supplementary material for: Long-term Associations of an Early Corrected Ventricular Septal Defect and Stress Systems of Child and Mother at Primary School Age
Source: Front Pediatr. 2018 Jan 15;5:293. doi: 10.3389/fped.2017.00293 (PMC5775274; doi:10.3389/fped.2017.00293)
Supplement: Supplementary file 3 [file table_3.PDF]

Table S3. ***Correlations between cortisol parameters and potential covariates***

	Child cortisol					Mother cortisol				
	Waking Cortisol (*n* = 35)	Bedtime Cortisol (*n* = 45)	CAR (*n* = 30)	Total release (*n* = 45)	Diurnal Slope (*n* = 35)	Waking Cortisol (*n* = 35)	Bedtime Cortisol (*n* = 47)	CAR (*n* = 30)	Total release (*n* = 47)	Diurnal Slope (*n* = 35)
SES	-.25	-.06	-.01	-.25	.03	.04	.11	-.05	-.03	.08
Age[a]	-.13	-.04	.28	.04	-.07	-.05	-.14	.36^{+}	-.01	.07
Child Sex	-.02	.23	-.13	-.01	.05	-.11	-.10	.35^{+}	-.00	.05
Child psychopathology (SDQ)	.24	.13	-.21	-.20	-.08	-.07	.39**	-.22	.12	.34*
Mother psychopathology (BSI)	.06	.26^{+}	.06	.17	.12	.02	.29*	.10	.37**	.22
Mother everyday stress (ESI)	.35*	.27^{+}	-.23	.39*	.04	-.06	.29*	.27	.47**	.18
School day	-.07	-.11	-.12	-.45**	-.05	.16	-.08	-.22	-.48**	-.24
Antibiotic intake[a]	.15	.01	-.26	.00	-.13	.27	-.16	-.28	-.03	-.25
Awakening time[a]	.07	.21	-.33^{+}	-.62**	-.04	.20	-.00	.54**	-.62**	-.10
Time Awakening - T1[a]	.15	.19	-.25	-.39*	.21	.22	-.24	-.25	-.38*	-.28
Time T1 - T5[a]	-.17	-.35*	.39*	.69**	.09	-.36*	-.03	.57**	.64**	.35*

Note: Correlations are Pearson product-moment-correlation coefficients. CAR = cortisol awakening response. Total release = total cortisol release throughout the day. SES = socioeconomic family status. SDQ = Strength and Difficulties Questionnaire (1). BSI = Brief Symptom Inventory (2). ESI = Everyday Stressors Index (3). [a]Correlations are calculated for the child or mother variable, respectively. $^{+}p < .10$, $*p < .05$. $**p < .01$.

References

1. Goodman R. Psychometric properties of the strengths and difficulties questionnaire. Journal of the American Academy of Child & Adolescent Psychiatry. 2001;40(11):1337-45.
2. Derogatis LR. BSI Brief Symptom Inventory. Administration, scoring, and procedures manual. 4 ed. Minneapolis, MN: National Computer Systems; 1993.
3. Hall L. Social support, everyday stressors, and maternal health. Unpublished doctoral dissertation; 1983.
