# Supplementary material for: Long-term Associations of an Early Corrected Ventricular Septal Defect and Stress Systems of Child and Mother at Primary School Age
Source: Front Pediatr. 2018 Jan 15;5:293. doi: 10.3389/fped.2017.00293 (PMC5775274; doi:10.3389/fped.2017.00293)
Supplement: Supplementary file 4 [file table_4.PDF]

Table S4. ***Correlations between characteristics of surgery or hospitalization and cortisol parameters within the VSD group***

	Child cortisol					Mother cortisol				
	Waking Cortisol (n = 17)	Bedtime Cortisol (n = 21)	CAR (n = 12)	Total release (n = 21)	Diurnal Slope (n = 17)	Waking Cortisol (n = 15)	Bedtime Cortisol (n = 23)	CAR (n = 12)	Total release (n = 23)	Diurnal Slope (n = 15)
Child age at surgery	.42^{+}	.06	-.20	.19	-.32	-.07	.12	-.27	.12	-.01
Duration of surgery	-.01	-.57**	-.14	-.52*	-.28	-.27	-.01	.07	-.04	-.28
Duration of connection to the heart-lung machine	.05	-.53*	-.25	-.51*	-.33	.24	.12	.07	.09	-.16
Duration of cardiac arrest	-.02	-.50*	-.11	-.39^{+}	-.23	.08	.17	.15	.13	-.11
Length of stay in the intensive care unit	-.46^{+}	.02	.31	-.30	.28	-.18	-.00	-.11	.09	.20
Length of stay in the hospital	-.35	-.28	.25	-.52*	.13	.-.06	-.31	-.18	-.19	-.16
Length of surgical scar	-.27	.33	-.12	.46*	.61**	-.02	.26	-.27	.08	.30

Note: Correlations are Spearman rank correlation coefficients because of the lacking normal-distribution of the surgery characteristics. CAR = cortisol awakening response. Total release = total cortisol release throughout the day. $^{+}p < .10$, $*p < .05$. $**p < .01$.
