# Supplementary material for: Long-term Associations of an Early Corrected Ventricular Septal Defect and Stress Systems of Child and Mother at Primary School Age
Source: Front Pediatr. 2018 Jan 15;5:293. doi: 10.3389/fped.2017.00293 (PMC5775274; doi:10.3389/fped.2017.00293)
Supplement: Supplementary file 5 [file table_5.PDF]

Table S5. ***Surgery characteristics predicting cortisol parameters ([A] bedtime cortisol, [B] total cortisol release, [C] diurnal cortisol slope) in children with an early corrected VSD: results of multiple regression models***

**[A] Bedtime cortisol**	Model 1				Model 2			
	B	95% CI	*t*	*p*	*B*	95% CI	*t*	*p*
Time T1 - T5[a]	0.05	[-0.15, 0.24]	0.51	.615	-0.05	[-0.23, 0.14]	-.51	.616
Duration of surgery					-0.01	[-0.01, -0.00]	-2.56*	.020
R^2	.01				.28			
F	0.26				3.44^{+}			
ΔR^2					.26			
ΔF					6.55*			
**[B] Total release**	Model 1				Model 2			
	B	95% CI	*t*	*p*	*B*	95% CI	*t*	*p*
Time T1 - T5[a]	2.03	[0.64, 3.42]	3.08	.007	1.45	[0.12, 2.78]	2.33*	.035
Schoolday[a]	1.22	[-3.32, 5.76]	0.57	.578	-0.21	[-4.47, 4.05]	-0.11	.917
Everyday stress[a]	0.18	[0.04, 0.32]	2.77	.013	0.09	[-0.05, 0.23]	1.35	.199
Duration of surgery					-0.01	[-0.05, 0,02]	-0.69	.502
Length of hospitalization					-0.32	[-0.59, -0.05]	-2.51*	.025
Length of surgical scar					0.61	[-0.29, 1.50]	1.45	.169
R^2	.62				.76			
F	9.05**				7.37**			
ΔR^2					.15			
ΔF					2.80^{+}			
**[C] Diurnal slope**	Model 1				Model 2			
	B	95% CI	*t*	*p*	*B*	95% CI	*t*	*p*
Time T1 - T5[a]	0.01	[-0.01, 0.04]	1.23	.236	0.01	[-0.01, 0.03]	0.91	.380
Length of surgical scar					0.01	[-0.00, 0.03]	2.00^{+}	.065
R^2	.09				.29			
F	1.52				2.92^{+}			
ΔR^2					.20			
ΔF					4.01^{+}			

Note. $n = 21$. Mother-rated everyday stress in Everyday Stressors Index (ESI; 1). [a]Specific covariates for predicted cortisol parameter. $^{+}p < .10$, $^{**}p < .01$.

References

1. Hall L. Social support, everyday stressors, and maternal health. Unpublished doctoral dissertation; 1983.
